# Metabolic Dysfunction-Associated Steatotic Liver Disease: Pathogenetic Links to Cardiovascular Risk

**DOI:** 10.3390/biom15020163

**Published:** 2025-01-22

**Authors:** Vlad Alexandru Ionescu, Gina Gheorghe, Nicolae Bacalbasa, Camelia Cristina Diaconu

**Affiliations:** 1 Faculty of Medicine, University of Medicine and Pharmacy Carol Davila Bucharest, 050474 Bucharest, Romania; vladalexandru.ionescu92@gmail.com; 2 Internal Medicine Department, Clinical Emergency Hospital of Bucharest, 105402 Bucharest, Romania; 3 Department of Visceral Surgery, Center of Digestive Diseases and Liver Transplantation, Fundeni Clinical Institute, 022328 Bucharest, Romania; nicolae_bacalbasa@yahoo.ro; 4 Department of Surgery, University of Medicine and Pharmacy Carol Davila Bucharest, 050474 Bucharest, Romania; 5 Academy of Romanian Scientists, 050085 Bucharest, Romania

**Keywords:** MASLD, cardiovascular risk, pathophysiology, myocardial ischemia, heart failure, atrial fibrillation

## Abstract

Metabolic dysfunction-associated steatotic liver disease (MASLD) is correlated with an increased cardiovascular risk, independent of other traditional risk factors. The mechanisms underlying this pathogenic link are complex yet remain incompletely elucidated. Among these, the most significant are visceral adiposity, low-grade inflammation and oxidative stress, endothelial dysfunction, prothrombotic status, insulin resistance, dyslipidemia and postprandial hyperlipemia, gut dysbiosis, and genetic mutations. Cardiovascular diseases are the leading cause of death in patients with MASLD. These patients have an increased incidence of coronary artery disease, carotid artery disease, structural and functional cardiac abnormalities, and valvulopathies, as well as arrhythmias and cardiac conduction disorders. In this review, we present the latest data on the association between MASLD and cardiovascular risk, focusing on the pathogenic mechanisms that explain the correlation between these two pathologies. Given the high rates of cardiovascular morbidity and mortality among patients with MASLD, we consider it imperative to raise awareness of the risks associated with this condition within the general population. Further research is essential to clarify the mechanisms underlying the increased cardiovascular risk linked to MASLD. This understanding may facilitate the identification of new diagnostic and prognostic biomarkers for these patients, as well as novel therapeutic targets.

## 1. Introduction

Metabolic dysfunction-associated steatotic liver disease (MASLD), previously referred to as non-alcoholic fatty liver disease (NAFLD), is defined as the correlation between steatotic liver disease (SLD) and one or more cardiometabolic risk factors in the absence of alcohol consumption (Table 1) [1]. The MASLD spectrum encompasses hepatic steatosis, metabolic dysfunction-associated steatohepatitis (MASH), liver fibrosis, liver cirrhosis, and hepatocellular carcinoma (HCC) [1].

MASLD is the most common chronic liver disease, affecting approximately 30% of the general population and over 50% of patients with diabetes [1,2]. From 1990 to 2019, the prevalence of this disease rose by 50.4%, increasing from 25.26% between 1990 and 2006 to 38% between 2016 and 2019 [2]. Moreover, the prevalence of MAFLD exhibits significant geographical variation, with the most affected regions being Latin America (44.37%), the Middle East (36.53%), North Africa (28.63–45.22%), South Asia (33.07%), Southeast Asia (33.07%), North America (31.20%), East Asia (29.71%), Asia Pacific (28.02%), and Western Europe (25.10%) [2].

Recent data from the United States’ United Network of Organ Sharing (UNOS) indicate that MASLD is the second most prevalent indication for liver transplantation [3]. Furthermore, MASH is the most rapidly expanding indication for liver transplantation in Western countries [4,5,6,7].

Approximately 10–25% of patients with MASLD may develop MASH, liver cirrhosis, or HCC; nonetheless, the primary cause of mortality in these patients is cardiovascular disease [8]. Therefore, these patients exhibit an elevated risk of developing ischemic heart disease, cardiomyopathies, chronic heart failure, cardiac arrhythmias, or heart conduction disorders [9,10,11]. For instance, stenoses are present in at least one coronary artery in approximately 50% of patients with MASLD [9]. Based on population studies that assessed patients with MASLD from 1990 to 2019, a recent meta-analysis reported a pooled mortality rate of 12.6 per 1000 person-years for all-cause mortality, 4.1 per 1000 person-years for cardiac-specific mortality, 2.8 per 1000 person-years for extrahepatic cancer-specific mortality, and 0.92 per 1000 person-years for liver-specific mortality [2]. A significant obstacle to the effective therapeutic care of these patients is the underdiagnosis of MASLD [12]. The factors contributing to this underdiagnosis are:

- Insufficient awareness of this condition among both patients and healthcare professionals.
- The disease’s initial asymptomatic nature.
- The absence of standardized diagnostic tools [12].

The annual economic burden of MASL was estimated to be approximately USD 103 billion in the United States of America (USA), EUR 27.7 billion in Germany, France, and Italy, and GBP 5.24 billion in Great Britain [9]. Furthermore, recent epidemiological data project that the costs required to care for these patients will rise to USD 1005 trillion in the USA and EUR 334 billion in Europe over the next decade [13].

The accelerated increase in the prevalence of MASLD and the high morbidity and mortality rates of these conditions underline the need for urgent strategies aimed at increasing awareness, as well as improving the diagnostic and therapeutic management of these patients. Our comprehensive narrative review presents the most recent data on MASLD and cardiometabolic risk. Consequently, we focused on presenting the mechanisms that explain the pathogenic link between the two types of conditions, while also highlighting a series of epidemiological, clinical, and paraclinical data regarding the correlation between these pathologies.

## 2. MASLD and Cardiovascular Diseases

**Figure 1:**
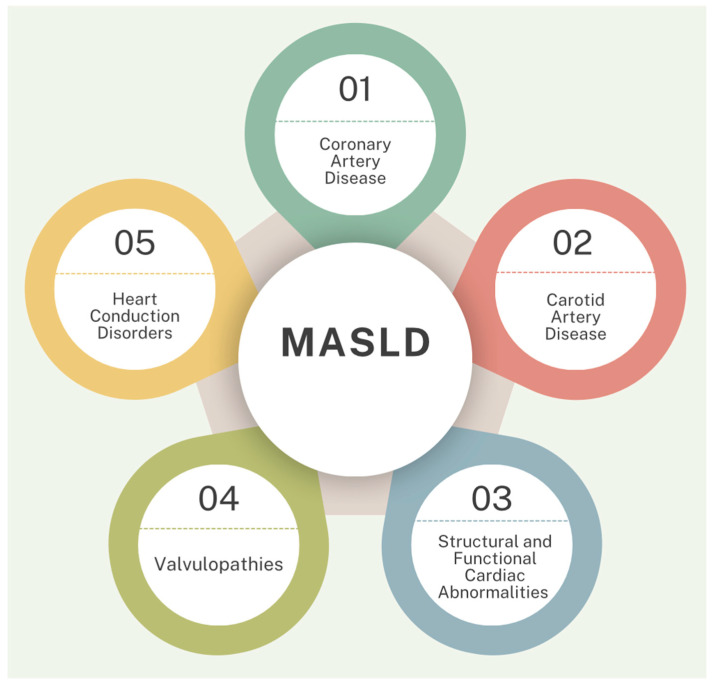
Cardiovascular disease in patients with MASLD [12].

Patients with MASLD have a high incidence of cardiovascular diseases, which are also the leading cause of death in this population (Figure 1) [14]. Thus, early diagnosis and proper management of this condition can significantly improve morbidity and mortality rates in these patients. The diagnosis of MASLD is based on the correlation of clinical data with laboratory and imaging investigations [15]. Patients with MASLD may exhibit mildly to moderately elevated aminotransferase levels [15]. Among these, alanine aminotransferase (ALT) is most closely associated with hepatic fat accumulation and is predominantly found in the cytoplasm of hepatocytes [16]. Patients with MASLD typically have higher ALT levels than aspartate aminotransferase (AST) levels, with an AST/ALT ratio (De Rittis ratio) < 1 [16]. This subunitary ratio has been independently correlated with MASLD risk and can serve as a non-invasive biomarker for identifying this metabolic condition [16]. Other biological changes suggestive of MASLD include elevated serum ferritin levels or transferrin saturation (observed in 6–11% of patients) and an increase in serum alkaline phosphatase levels, which may reach 2–3 times the upper normal limit [15]. Key imaging findings in MASLD patients include increased hepatic echogenicity on ultrasound, increased fat signal on MRI, and decreased hepatic attenuation on CT [15]. The most commonly used diagnostic method for MASLD remains abdominal ultrasound, with a sensitivity of 89% and a specificity of 93% [15].

MASLD is an independent risk factor for ASCVD [12]. Both patients with MASLD and those with alcoholic liver disease (ALD) exhibit elevated coronary calcium scores (CCS) [12]. CCS is an independent predictive marker for cardiovascular events, reflecting reduced vascular compliance, extensive atherosclerotic lesions, abnormal vasomotor responses, and impaired coronary perfusion [14,17,18]. Kang et al. demonstrated in a recent study that patients with MASLD have an increased risk of coronary artery calcification, independent of other risk factors such as age, sex, smoking, or diabetes [19]. Vega et al. reported in a study including 113 patients with symptoms suggestive of coronary artery disease that 52% had MASLD [20]. A meta-analysis of 15 studies including over 10 million individuals showed increased all-cause and cardiovascular mortality in patients with MASLD [21]. Another recent meta-analysis of 4725 patients with MASLD reported a direct proportional relationship between the degree of hepatic fibrosis and subclinical atherosclerosis [22]. Furthermore, patients with MASLD have a 60% higher risk of acute myocardial infarction and stroke compared to those without this metabolic condition [23].

Coronary collateral circulation is an important protective mechanism for myocardial cells in the event of an occlusion in a coronary artery [24]. The development of this collateral circulation is influenced by several factors, with diabetes mellitus being a well-established condition associated with poor coronary collateral circulation [24]. However, this phenomenon has also been observed in patients with insulin resistance [25]. Arslan et al. demonstrated an association between MASLD and poor coronary collateral circulation in patients without diabetes [25]. The pathogenic mechanisms that may explain this phenomenon include the proinflammatory and pro-thrombotic states, endothelial dysfunction, and accelerated atherogenesis commonly found in patients with MASLD [24].

Current evidence supports the association of MASLD with both subclinical cardiovascular conditions (e.g., CCS, abdominal aortic calcification, increased carotid intima-media thickness [CIMT], endothelial dysfunction, and arterial stiffness) and clinical conditions (e.g., acute coronary syndrome, coronary artery disease, and ischemic stroke) [26]. Increased CIMT is independently associated with both the degree of hepatic steatosis and subclinical atherosclerosis, as well as the risk of acute coronary syndrome or ischemic stroke [26]. A recent meta-analysis demonstrated a higher prevalence of subclinical atherosclerosis, assessed by CIMT and CCS, in patients with MASLD, independent of other common cardiometabolic risk factors [27]. These findings were reported in both Western and Asian populations [27]. Bhatia et al. showed in a randomized, double-blind, placebo-controlled study that reducing the severity of hepatic steatosis correlates with the attenuation of CIMT progression, a validated and widely accepted screening tool for cardiovascular risk assessment [28]. Another study demonstrated a direct proportional relationship between the reduction in fatty liver index (FLI) and improvements in both CIMT and flow-mediated dilation (FMD) [29].

There is now clear evidence linking MASLD to a series of structural and functional cardiac abnormalities, both in the presence and absence of other features of metabolic syndrome [12]. Most studies have reported a significant association between MASLD and structural and functional alterations of the left ventricle, including left ventricular hypertrophy and diastolic dysfunction [30,31,32,33]. Gohil et al. recently reported a significant association between MASLD and left ventricular diastolic dysfunction, emphasizing its higher prevalence among non-obese MASLD patients [33]. These findings are consistent with those of Cong et al., who observed a higher prevalence of an E/A ratio < 1 in non-obese MASLD patients in a cross-sectional study [32]. Another recent study demonstrated a direct proportional relationship between the severity of hepatic steatosis and the severity of left ventricular diastolic dysfunction, even after adjusting for potential confounding factors [34]. The CARDIA (Coronary Artery Risk Development in Young Adults) study highlighted an association between MASLD and an increased incidence of abnormal left ventricular geometry, left ventricular hypertrophy, and worsening myocardial strain, independent of traditional heart failure risk factors or changes in BMI [35]. This study also identified MASLD as a risk factor for long-term left ventricular remodeling [35]. These findings are further supported by Hung et al., who demonstrated a direct proportional relationship between MASLD severity and the incidence of left ventricular hypertrophy and abnormal QTc intervals [36]. They advocate for regular electrocardiographic and echocardiographic evaluations in MASLD patients to enable the early detection of arrhythmias or left ventricular hypertrophy [36].

Aortic valve sclerosis and mitral annular calcification are identified in over 20% of adults aged 65 years and older [37]. However, patients with MASLD have a 32% higher risk of being diagnosed with aortic valve sclerosis compared to those without MASLD [38]. Mantovani et al. reported that MASLD is an independent predictor for mitral and aortic valve calcification in patients with diabetes mellitus [39]. The pathogenic mechanisms involved include oxidative stress and a proinflammatory state [40]. Oxidized dicarboxylic acids (derived from medium-chain lipid peroxides) can bind to calcium and induce calcification in smooth muscle cells [40].

Structural and functional cardiac abnormalities ultimately lead to chronic heart failure [41,42,43]. MASLD is associated with a 1.5-fold higher risk of developing heart failure, independent of diabetes mellitus, hypertension, and other common cardiovascular risk factors [42]. Additionally, these patients are predisposed to progression towards heart failure with preserved ejection fraction (HFpEF) [41]. Furthermore, a recent study reported a direct proportional relationship between MASLD severity and unfavorable outcomes in patients hospitalized for decompensated HFpEF episodes [44].

Patients with MASLD have an increased risk of developing arrhythmias and cardiac conduction disorders [45,46,47]. The most prevalent among these are atrial fibrillation, prolonged QT interval, and atrioventricular blocks [45,46,47]. A meta-analysis involving 364,919 MASLD patients reported a correlation between MASLD and an elevated risk of atrial fibrillation, particularly in patients with coexisting diabetes mellitus [48]. Markus et al. demonstrated a relationship between moderate elevations in liver enzymes and an increased prevalence of atrial fibrillation [49]. The proposed pathogenic mechanisms for this association include elevated levels of proinflammatory, pro-coagulant, and pro-fibrinogenic factors, which induce structural and electrical remodeling of the atria [49]. Additionally, Mantovani suggested that hyperuricemia might play a role in the link between MASLD and atrial fibrillation [48]. Mahfouz et al. proposed that the increased thickness of the interatrial septum and the left atrial stiffness index could explain the higher prevalence of atrial fibrillation in MASLD patients [50]. There is also evidence supporting the role of epicardial and pericardial adipose tissue expansion in the development of cardiac arrhythmias in MASLD patients [51].

Regarding the relationship between MASLD and atrioventricular blocks or fascicular blocks, it has been shown to be bidirectional [8]. On the one hand, MASLD increases the risk of cardiac block approximately threefold, independent of other common risk factors. On the other hand, young patients with a newly diagnosed right bundle branch block, without other cardiac pathology, have an increased risk of also having MASLD [52,53]. Additionally, there is a direct proportional correlation between the severity of MASLD and the risk of a prolonged QTc interval in the general population, independent of coexisting diabetes mellitus [54,55,56].

## 3. Pathophysiology of MASLD and Cardiovascular Diseases

**Figure 2:**
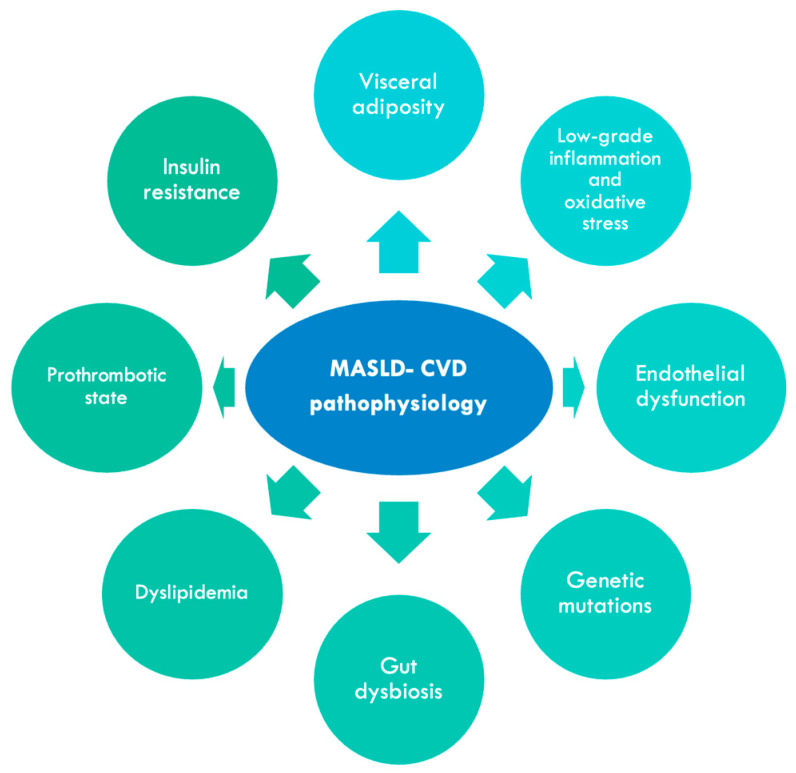
Pathophysiology of MASLD and cardiovascular diseases.

The pathogenic mechanisms underlying the complex relationship between MASLD and cardiovascular diseases remain incompletely elucidated. These mechanisms include visceral adiposity, low-grade inflammation and oxidative stress, endothelial dysfunction, insulin resistance, dyslipidemia, postprandial hyperlipidemia, intestinal dysbiosis, and genetic mutations (Figure 2) [8].

### 3.1. Visceral Adiposity

Obesity is a common risk factor for both MASLD and cardiovascular diseases. Recent studies indicate that the global prevalence of steatotic liver disease (SLD) in adults of normal weight varies between 3% and 30% [57,58]. Furthermore, normal-weight individuals with SLD exhibit a cardiovascular disease risk comparable to that of obese individuals with SLD [57]. Data from recent decades suggest that central obesity is more closely associated with metabolic disorders than an increased BMI [59]. Thus, individuals with central adipose tissue deposition but a normal BMI represent a highly susceptible subgroup for developing metabolic abnormalities, particularly MASLD [59].

Visceral adipose tissue functions as an endocrine organ, secreting hormones, adipokines, and cytokines that play a role in the development of insulin resistance and chronic inflammation [60]. Abdominal adiposity is associated with adiposopathy, characterized by enlarged, predominantly dysfunctional adipocytes and a hyperlipolytic state, exposing the liver to high concentrations of free fatty acids (FFA) and glycerol [59]. Numerous studies have reported that the release of FFA from visceral adipose tissue is more significant than total body fat content in the development and progression of MASLD [59].

These changes lead to several disruptions in the hepatic metabolism, such as an exacerbation of hyperinsulinemia and increased triglyceride production [61]. Additionally, hepatic fat accumulation has several pathological consequences, including the generation of cytotoxic lipid species, inflammasome activation, and oxidative stress [62,63,64].

MASLD is associated with ectopic fat deposition in other structures, such as the epicardium and pericardium [8]. Under physiological conditions, epicardial adipose tissue has important functions for the myocardium, including thermogenic, mechanical, and metabolic support [65]. Additionally, perivascular fat exhibits vasoactive properties [66]. However, excess epicardial adipose tissue leads to myocardial tissue hypertrophy, fibrosis, and reduced adiponectin synthesis because of the increased release of inflammatory factors, such as tumor necrosis factor-α, interleukin-6, and interleukin 1-β [67]. The Framingham Heart Study identifies epicardial adipose tissue as an independent cardiovascular risk factor [68]. Furthermore, pericardial fat volume has been shown to correlate with the prevalence of atrial fibrillation and serves as an independent risk factor for major cardiovascular events [69,70]. Lipid deposition in cardiomyocytes (myocardial steatosis) contributes locally to myocardial dysfunction, while fat surrounding the renal arteries is associated with poor blood pressure control and progression to chronic kidney disease [71,72,73,74].

Visceral adipose tissue secretes numerous adipokines, including leptin, omentin, adiponectin, visfatin, resistin, and apelin (Table 2) [75]. An elevation in BMI is associated with hyperleptinemia and leptin resistance, leading to reduced energy consumption, hyperinsulinemia, and hyperlipidemia [76,77]. Additionally, visceral adipose tissue volume is correlated with serum leptin levels, which serve as a predictive marker for cardiovascular risk [78]. Visceral fat accumulation is correlated with reduced plasma levels of adiponectin, which subsequently impairs fatty acid oxidation and disrupts the body’s energy homeostasis [79,80,81,82]. Unlike other adipokines, which are abundant in both visceral and subcutaneous adipose tissue, adiponectin is primarily produced by adipose tissue in the bone marrow [82]. Low levels of adiponectin are associated with chronic inflammation, atherosclerosis, type 2 diabetes, and obesity [83]. Omentin enhances insulin action without affecting basal glucose transport [84]. Other adipokines with protective effects include vaspin, which inhibits reactive oxygen species, and apelin, which promotes cholesterol efflux [85]. All these adipokines have reduced serum levels in overweight patients [84,85].

### 3.2. Low-Grade Inflammation and Oxidative Stress

Patients with MASLD exhibit a low-grade inflammatory syndrome, primarily explained by the secretion of proinflammatory cytokines from adipose tissue and intestinal dysbiosis characteristic of these patients [86]. A recent study demonstrated that macrophage scavenger receptor 1 (MSR1) induces a proinflammatory response through the c-Jun N-terminal Kinases (JNK) signaling pathway in mice fed a high-fat and high-cholesterol diet [87]. This response is characterized by increased production of proinflammatory and proatherogenic cytokines, such as interleukin-6 (IL-6) and tumor necrosis factor (TNF), both involved in the pathogenesis of cardiovascular diseases [87]. Another study showed that IL-6 deletion attenuates left ventricular hypertrophy and dysfunction, highlighting the essential role of this cytokine in cardiomyocyte hypertrophy [88]. IL-6 activates the Janus Kinase/signal transducers and activators of the transcription (JAK/STAT) signaling cascade and regulates the transcription of acute-phase reactants such as C-reactive protein (CRP) and serum amyloid A (SAA), which further amplify the hepatic inflammatory response [89]. Moreover, serum levels of IL-6 and highly sensitive C-reactive protein (hsCRP) have been independently associated with cardiovascular disease risk [90,91,92]. Elevated levels of proinflammatory cytokines, such as TNF-alpha and IL-6, correlate with the initiation and progression of atherosclerosis by promoting endothelial dysfunction, plaque instability, and endothelial wall damage [89].

A diet rich in lipids and carbohydrates alters the composition of gut microbiota, increasing lipopolysaccharide production and activating proinflammatory pathways [93]. A recent meta-analysis demonstrated that probiotic and prebiotic therapy can improve inflammation, insulin resistance, dyslipidemia, and liver injury in patients with MASLD by enhancing the intestinal barrier, preventing the formation of hepatotoxic metabolites, and modulating the immune system [94].

The secretion of micro ribonucleic acids (miRNAs) is altered in patients with MASLD compared to healthy individuals. The steatotic liver increases the release of hepatocyte-derived extracellular vesicles containing miRNAs with proinflammatory and proatherosclerotic roles [86]. These include miRNA-30a-3p, which promotes atherosclerosis by inhibiting ABC1-mediated cholesterol efflux, and miRNA-1, which induces endothelial inflammation by activating the nuclear factor-kappa B (NF-kB) signaling pathway [95,96].

Other pathogenic hypotheses highlight the proinflammatory role of specific macrophage populations found in the liver [97]. Elevated levels of FFAs and triglycerides lead to increased lipid oxidation, the generation of reactive oxygen species (ROS), and lipotoxicity, resulting in cellular injury [98]. These processes trigger the release of cytokines and the activation of Kupffer cells, a subset of hepatic macrophages that recruit monocytes and other immune cells from the bloodstream, thereby exacerbating the inflammatory process [98]. Oxidative stress is closely linked to the pathophysiology of MASLD and cardiovascular diseases [99]. Excess fat accumulation in the liver leads to mitochondrial dysfunction and increased production of ROS, promoting inflammation and fibrosis [99]. This process can exacerbate insulin resistance, a hallmark of MASLD [99]. Moreover, patients with MASLD exhibit reduced levels of glutathione, an antioxidant primarily produced in the liver [100]. Decreased hepatic glutathione levels can impair the redox state not only in the liver but also in the heart and other organs [100]. Oxidative stress promotes atherosclerosis and may impair cardiovascular function, contributing to the development of hypertension, myocardial infarction, and heart failure [101]. Lee et al. demonstrated that oxidative stress correlates with a higher incidence of metabolic and cardiovascular diseases [102].

### 3.3. Endothelial Dysfunction

Endothelial dysfunction is the initial stage of the atherosclerotic process, and plays a critical role in the progression of cardiovascular diseases [103]. It results from the interplay between oxidative stress, vascular inflammation (stimulated by lipoproteins such as apolipoprotein C3), and selective insulin resistance at the vascular level [103]. A key feature of endothelial dysfunction is the reduction in serum nitric oxide levels, a substance with vasodilatory properties [103]. This reduction leads to vasoconstriction, increased vascular resistance, and elevated blood pressure [103].

**Figure 3:**
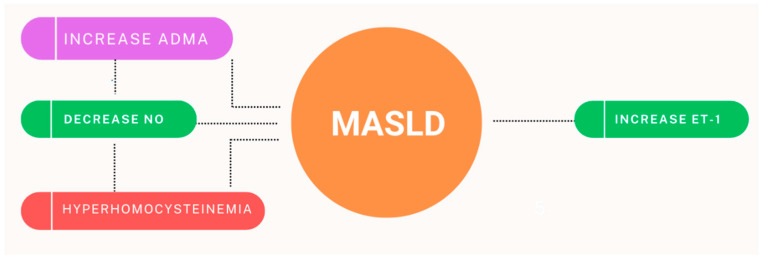
Pathogenic mechanisms implicated in the onset of endothelial dysfunction in patients with MASLD (ADMA—asymmetric dimethylarginine, NO—nitric oxide, ET-1—endothelin-1) [104,105,106,107,108].

Patients with MASLD exhibit elevated levels of asymmetric dimethylarginine (ADMA), a natural antagonist of nitric oxide synthesis [104]. Increased ADMA levels are attributed to oxidative stress, a proinflammatory state, and enhanced methylation in the liver [105]. Additionally, these patients often present with hyperhomocysteinemia, which further reduces nitric oxide production [106]. Hyperhomocysteinemia also contributes to oxidative stress and increased platelet activation [107]. Beyond the reduction in nitric oxide levels, the heightened vasoconstriction observed in patients with MASLD can also be explained by elevated levels of endothelin-1, induced by the proinflammatory state and oxidative stress (Figure 3) [108].

The phenotypic changes induced by endothelial dysfunction include an increased expression of cell adhesion molecules, elevated chemokine secretion, increased cell permeability, oxidation of low-density lipoproteins, platelet activation, and proliferation of vascular smooth muscle cells [106].

### 3.4. Insulin Resistance

Under physiological conditions, insulin promotes glucose absorption by tissues and its conversion into glycogen, while simultaneously suppressing hepatic glucose synthesis. Insulin resistance, a central feature of MASLD, is characterized by impaired mechanisms of glucose homeostasis regulation mediated by this hormone [109].

Insulin resistance in the skeletal muscle reduces glycogen synthesis and increases de novo hepatic lipogenesis and triglyceride synthesis [110,111]. These processes lead to the development of atherogenic dyslipidemia, even in normal weight individuals [86]. Increased de novo lipogenesis results in the accumulation of diacylglycerols (intermediates in the biosynthesis of triacylglycerols) in the liver [112]. Elevated diacylglycerol levels lead to the translocation of protein kinase Cε (PKCε) to the plasma membrane, where it interacts with the kinase domain of the insulin receptor, thereby inhibiting the phosphorylation of insulin receptor substrate 2 (IRS-2) [112]. The result is a reduced insulin response, accompanied by increased plasma glucose and insulin levels [113]. Insulin also activates enzymes involved in fatty acid synthesis, further promoting de novo hepatic lipogenesis and triglyceride synthesis [114]. Under normal conditions, the liver synthesizes triglycerides from FFAs and packages them into very low-density lipoprotein (VLDL) particles for storage or export [115]. Insulin resistance disrupts this process, leading to lipid accumulation in hepatocytes and exacerbating hepatic steatosis [115]. Additionally, lipid imbalance promotes the development of atherosclerosis, significantly increasing cardiovascular risk [115,116,117]. Conversely, chronic hyperglycemia induces oxidative stress and promotes a persistent inflammatory response [116].

The correlation between cell membrane composition and insulin resistance is a key factor in the pathogenesis of MASLD and cardiovascular diseases [118]. Alterations in the lipid composition of cell membranes, characterized by the accumulation of saturated fatty acids and cholesterol, are associated with an increased synthesis of proinflammatory factors such as interleukin-6 (IL-6), tumor necrosis factor (TNF), adhesion molecules, and C-reactive protein [119]. These changes contribute to cell membrane stiffness, leading to a reduction in the number of insulin receptors and their affinity for insulin [118]. Future studies are needed to identify mechanisms that could reduce cell membrane stiffness, improve cell membrane fluidity, and subsequently enhance the number of insulin receptors and their affinity for insulin.

Colantoni et al. demonstrated in a recent study that the Homeostatic Model Assessment for Insulin Resistance (HOMA-IR) and Lipid Accumulation Product (LAP) tests have the highest accuracy in detecting MASLD [120]. The same authors suggested the utility of insulin resistance markers for cardiovascular risk stratification in patients with MASLD [120]. A major implication of hepatic insulin resistance is its connection to vascular insulin resistance and endothelial dysfunction [121].

Since MASLD is considered a hepatic manifestation of metabolic syndrome, it is closely associated with type 2 diabetes mellitus (T2DM) [122]. A recent meta-analysis including nearly 2 million patients reported a MASLD prevalence of 65.04% among individuals with T2DM [123]. On the other hand, the presence of hepatic fibrosis is associated with a 2.95-fold increased risk of developing T2DM and is considered an independent predictor of the condition. The pathogenic mechanisms of MASLD and T2DM are complex, with insulin resistance being recognized as a central factor in the development of both diseases [122].

### 3.5. Dyslipidemia and Postprandial Hyperlipemia

A key feature of MASLD is the disruption of lipid metabolism homeostasis [124]. This includes:

- Increased hepatic fatty acid uptake;
- Suppressed fatty acid oxidation;
- De novo lipogenesis;
- Increased secretion of very-low-density lipoproteins (VLDL);
- Reduced secretion of high-density lipoprotein cholesterol (HDL-c) [124].

In the liver, fatty acids undergo either oxidation or esterification to form triglycerides, which are stored or exported into the circulation as VLDL particles [125]. Hepatic steatosis occurs when the rate of triglyceride synthesis exceeds their elimination into the circulation [125]. This imbalance can result from excessive dietary intake or increased de novo lipogenesis [125].

**Figure 4:**
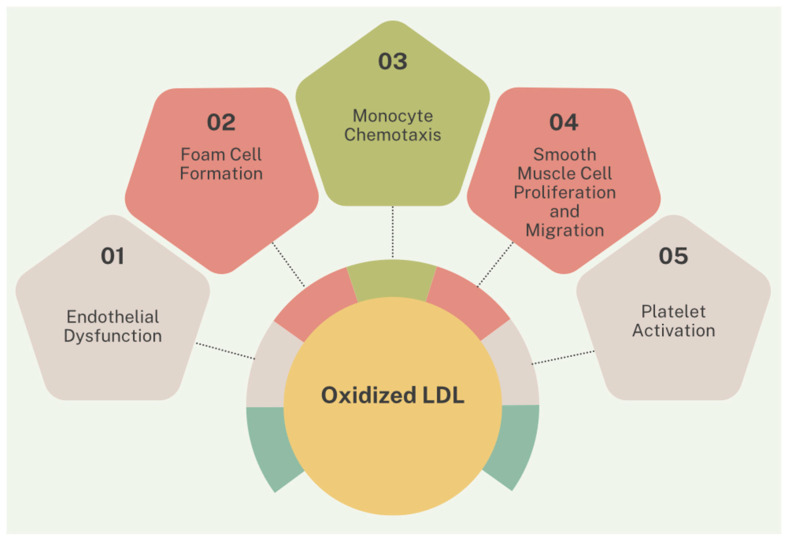
The mechanisms by which oxidized LDL-c contribute to the instability and rupture of the atheromatous plaque.

Dyslipidemia is a key factor in the development of atherosclerosis and cardiovascular diseases. Low-density lipoprotein cholesterol (LDL-c) particles can penetrate the endothelium, where they undergo oxidation and trigger inflammatory pathways essential for the development of atherosclerotic plaques [126]. Oxidized LDL-c is involved in endothelial dysfunction, foam cell formation (through macrophage absorption of LDL-c), monocyte chemotaxis, smooth muscle cell proliferation and migration, and platelet activation. These processes collectively culminate in plaque instability and rupture (Figure 4) [126,127,128].

Postprandial hyperlipemia is defined as a postprandial increase in serum triglyceride levels, a process involved in the pathogenesis of postprandial atherosclerotic disease [129,130]. Elevated serum triglycerides levels reflect an increase in triglyceride-rich lipoproteins (TGLs) such as chylomicrons and VLDL [129]. These biological changes are observed in patients with metabolic syndrome, type 2 diabetes, chronic kidney disease, familial combined hyperlipidemia, or familial type II hyperlipoproteinemia [129,131,132].

**Figure 5:**
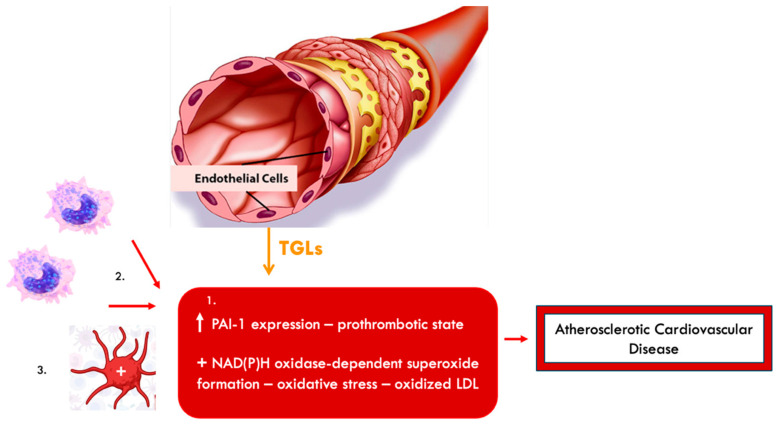
Mechanisms by which triglyceride-rich lipoproteins (TGLs) contribute to the development of atherosclerotic cardiovascular disease. 1. Prothrombotic state through increased expression of plasminogen activator inhibitor-1 (PAI-1) and induction of oxidative stress via NAD(P)H oxidase-dependent superoxide formation. 2. Migration of monocytes to endothelial cells. 3. Platelet activation.

Postprandial hyperlipemia is closely associated with postprandial hyperglycemia, and insulin resistance can induce or exacerbate both conditions [129]. TGLs stimulate monocyte migration and adhesion to endothelial cells [129]. They also contribute to inflammatory syndrome, platelet activation, and endothelial dysfunction by activating transcription factors like NF-kB in endothelial cells and monocytes [129]. In dysfunctional endothelial cells, the expression of plasminogen activator inhibitor-1 (PAI-1) increases, reducing fibrinolysis and promoting a prothrombotic state [129,133]. TGLs also stimulate the formation of NAD(P)H oxidase-dependent superoxide via the activation of the lectin-like oxidized LDL-c receptor-1. This results in the oxidation of LDL-c within the endothelium, driving atherosclerosis development (Figure 5) [129].

### 3.6. Gut Dysbiosis

Recent research has revealed complex connections between gut dysbiosis, MASLD, and cardiovascular risk [134]. Gut dysbiosis contributes to the development and progression of MASLD through the dysregulation of the gut–liver axis, comprising systemic circulation, portal circulation, and bile ducts [134]. This leads to increased intestinal permeability and translocation of microbial metabolites to the liver, causing secondary inflammation [134].

The gut microbiota also influences bile acid composition (via the enterohepatic circulation), lipid and glucose metabolism, and energy balance [135,136]. Intestinal microorganisms convert undigested polysaccharides into monosaccharides and dietary fibers into short-chain fatty acids (SCFAs), both of which provide energy support for host cells [137]. SCFAs play a role in regulating energy metabolism and the immune system [137]. They also promote colonic motility, protect the intestinal mucosal barrier, regulate carbohydrate and lipid metabolism, and improve the absorption of electrolytes and nutrients. At the same time, SCFAs exhibit anti-inflammatory and antitumor activities [137].

Systemic inflammation secondary to the translocation of microbial metabolites into the systemic circulation can promote endothelial dysfunction and atherosclerosis, thereby increasing cardiovascular risk [138]. Intestinal bacteria also convert dietary choline and L-carnitine into trimethylamine (TMA) [139]. TMA is subsequently metabolized in the liver into trimethylamine N-oxide (TMAO), which contributes to endothelial dysfunction by modulating the levels of vasoregulatory substances (including nitric oxide) and cellular adhesion molecules [139]. In a multicenter study, Li et al. demonstrated that elevated plasma TMAO levels in patients with acute coronary syndromes could predict 7-year mortality and the risk of major adverse cardiac events at 30 days and 6 months [140].

Indoxyl sulfate, another intestinal metabolite, has been shown to exert proinflammatory and pro-oxidative effects, influencing blood pressure values [141]. Simultaneously, specific microbial profiles and intestinal metabolites, such as SCFAs, lipopolysaccharides (LPS), bile acids, choline, and TMA, have been correlated with liver disease severity and the stage of hepatic fibrosis [142].

Splanchnic circulation congestion, intestinal wall edema, and increased intestinal permeability facilitate the translocation of bacteria and their metabolites into systemic circulation, thereby exacerbating the proinflammatory state associated with cardiovascular diseases [137].

Among the specific effects of SCFAs on the cardiovascular system are the regulation of gut barrier integrity with anti-inflammatory effects, the modulation of lipid metabolism, and influence on insulin sensitivity [143,144]. For instance, butyrate and propionate can reduce blood pressure and ameliorate ischemia/reperfusion injuries, thereby decreasing the risk of coronary artery disease. Additionally, acetate may play a positive role in regulating hypertension and preventing atherosclerosis [143]. These SCFAs have been shown to have significantly lower levels in patients with MASLD, potentially contributing to the development of this condition and, subsequently, to an increased cardiovascular risk [144].

### 3.7. Genetic Mutations

Certain genetic mutations are associated with both MASLD and cardiovascular diseases, representing potential pathogenic components mediating both conditions [145]. The genes implicated in this pathogenic link encode the synthesis of the following biomolecules:

- Apolipoprotein C-3 (*APOC3*);
- Apolipoprotein E (*APOE*);
- Insulin receptor substrate-1 (*IRS-1*);
- Phosphatidylethanolamine N-methyltransferase (*PEMT*);
- Tribbles homolog 1 protein (*TRIB1 gene*);
- Tumor necrosis factor-alpha (TNF-alpha);
- Interleukin 6 (IL-6);
- Patatin-like phospholipase domain-containing protein 3 (*PNPLA3*);
- Transmembrane 6 superfamily 2 (*TM6SF2*);
- Glucokinase regulatory protein (*GCKR*) [145].

*APOC3* is a key regulator of plasma triglyceride levels [146]. It reduces lipoprotein lipase activity and inhibits triglyceride hydrolysis in VLDL and chylomicrons, thereby increasing plasma triglyceride levels and cardiovascular risk [146]. The genetic variants *C482T* and *T455C* are associated with a 30% higher serum APOC3 level and a 60% higher serum triglyceride level, respectively [147]. Additionally, 38% of carriers of these genetic variants are diagnosed with MASLD [147].

*APOE* facilitates the clearance of triglyceride-rich lipoproteins from the bloodstream [99]. It also influences vascular function through mechanisms such as the inflammatory response and platelet aggregation [148]. The prevalence of the *APOE ε3* allele has been found to be increased in patients with MASLD [149]. Conversely, the *APOE ε2* allele has a protective role against MASLD and exerts a hypolipidemic effect, being associated with lower LDL-c levels and higher HDL-c levels [150,151]. In contrast, the *APOE ε4* allele has been shown to increase cardiovascular risk by elevating carotid intima-media thickness (CIMT) as well as serum levels of LDL-c, lipoprotein(a), and apolipoprotein B [152,153,154].

*PEMT* catalyzes the conversion of phosphatidylethanolamine to phosphatidylcholine, a key component in VLDL synthesis [155]. The *V175Met* mutation is associated with reduced PEMT activity, an increased risk of progression to MASH, and lower levels of cardioprotective lipids such as LPC 16:0 and LPC 20:4 [156].

The genetic polymorphism of *IRS-1* leads to reduced *IRS-1* expression, resulting in an increased visceral fat-to-subcutaneous fat ratio, insulin resistance, hyperlipemia, and decreased adiponectin levels [153]. The final outcome is increased cardiovascular risk [153]. Additionally, *Gly972Arg* polymorphism is associated with impaired insulin receptor activity and increased MASLD severity [154].

*TRIB1* reduces plasma triglycerides levels and is involved in maintaining the balance of phagocytosis, M2 macrophage differentiation, and inhibition of inflammatory chemotaxis [157]. The *TRIB1 rs2954021* allele has been associated with elevated alanine transaminase (ALT) levels and an increased likelihood of a MASLD diagnosis [157]. The same allele has also been linked to an elevated risk of coronary heart disease and ischemic stroke [158].

The *PNPLA3 rs738409G* allele encodes the PNPLA3 protein, which reduces triglyceride hydrolysis and VLDL production, thereby lowering cardiovascular risk [159]. However, the inhibition of PNPLA3 increases the risk of major cardiovascular diseases, such as acute myocardial infarction or heart failure [159].

The *TM6SF2 E167K* genetic variant has been associated with hepatic triglyceride content and cardiovascular risk [160]. Recent studies have highlighted the role of TM6SF2 in promoting steatosis, hepatic fibrosis, and hepatocarcinogenesis [161,162]. Surprisingly, however, the *TM6SF2 E167K* variant has demonstrated a cardioprotective effect, primarily attributed to lower triglyceride and LDL-c levels [163]. Additionally, MASLD patients carrying the *TM6SF2 E167K* variant have shown lower serum levels of CRP, a validated biomarker for atherosclerotic cardiovascular disease (ASCVD) [164].

*GCKR* is involved in regulating glucose metabolism and de novo lipogenesis [165]. The *rs1260326-T* variant is associated with increased plasma levels of apolipoprotein B and a higher risk of MASLD [166]. Similarly, the *rs789904-T* variant is linked to hepatic steatosis and an increased cardiovascular risk because of elevated serum levels of triacylglycerols and VLDL, along with reduced HDL-c levels [166].

### 3.8. Prothrombotic State

**Figure 6:**
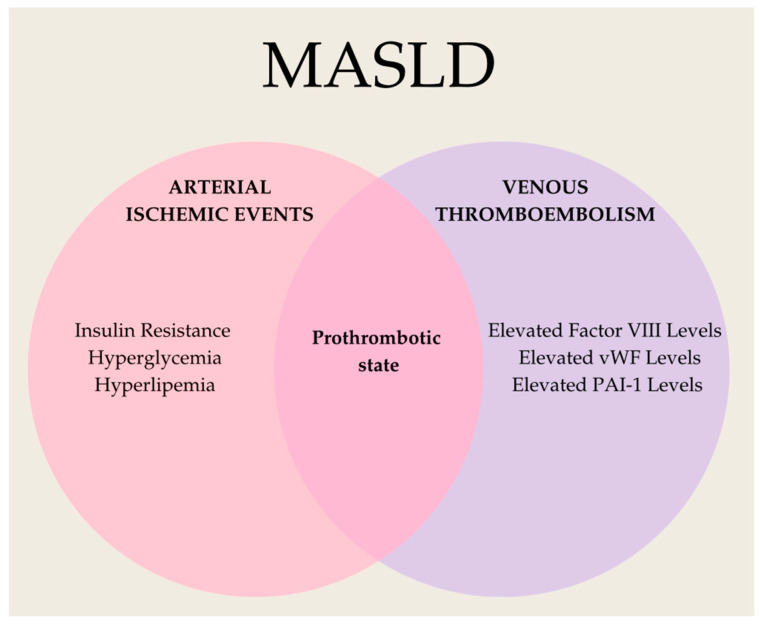
Pathogenic mechanisms increasing the risk of arterial ischemic events and venous thromboembolism in patients with MASLD (vWF—von Willebrand factor, PAI-1—plasminogen activator inhibitor-1).

The predominant vascular complications in patients with MASLD are arterial ischemic events [167]. Insulin resistance, hyperglycemia, and hyperlipemia play crucial roles in their development [167]. Additionally, patients with MASLD have been shown to be at a higher risk of venous thromboembolism, driven by hypercoagulability and reduced fibrinolysis [167]. The key factors linking MASLD to venous thromboembolism include elevated levels of factor VIII, von Willebrand factor, and PAI-1 (Figure 6) [167].

PAI-1 levels do not seem to correlate with total body fat mass; instead, they are associated with hepatic lipid accumulation [168]. Additionally, inflammation, as evidenced by elevated CRP levels, promotes PAI-1 activity and enhances monocyte–endothelium interactions [168]. Furthermore, inflammation is associated with reduced levels of antithrombin, a natural anticoagulant [168]. Endothelial cells actively contribute to the inflammatory and procoagulant state by producing several molecules, including von Willebrand factor, factor VIII, and fibrinogen [17]. The levels of these molecules are significantly higher in patients with MASLD, serving as predictive factors for both arterial and venous thrombosis [18].

## 4. Conclusions

In conclusion, MASLD is associated with an increased risk of cardiovascular diseases, independent of other traditional risk factors. The pathogenic mechanisms underlying this correlation are complex but remain incompletely elucidated. Given the high rates of cardiovascular morbidity and mortality among these patients, future studies are warranted to clarify the mechanisms leading to increased cardiovascular risk in MASLD. Such research could facilitate the development of diagnostic and prognostic biomarkers for these patients and the identification of new therapeutic targets. Additionally, we emphasize the need for informational campaigns aimed at raising awareness about the necessity of cardiovascular screening in patients with MASLD.

## Figures and Tables

**Table 1:** Cardiometabolic risk factors in defining MASLD [1].

Cardiometabolic Risk Factors	
Overweight/Obesity	BMI ≥ 25 kg/m^2^ (≥23 kg/m^2^—Asians) Waist circumference: ➢ ≥80 cm in women and ≥94 cm in men (Europeans) ➢ ≥80 cm in women and ≥90 cm in men (Chinese and South Asians) ➢ ≥90 cm in women and ≥85 cm in men (Japanese)
Prediabetes/type 2 diabetes	Prediabetes: HbA_1c_ 5.7–6.4% or fasting plasma glucose 100–125 mg/dL or 2-h plasma glucose during OGTT 140–199 mg/dL. Type 2 diabetes: HbA_1c_ ≥ 6.5% or fasting plasma glucose ≥ 126 mg/dL or 2-h plasma glucose during OGTT ≥ 200 mg/dL. Treatment for type 2 diabetes.
Plasma triglycerides	≥150 mg/dL Lipid-lowering treatment
HDL-cholesterol	≤50 mg/dL in women and ≤39 mg/dL in men Lipid-lowering treatment
Blood pressure	≥130/85 mmHg Treatment for hypertension

Legend: BMI—body mass index, HbA_1c_—glycated hemoglobin, HDL—high-density lipoprotein, OGTT—oral glucose tolerance test.

**Table 2:** Adipokines proven to influence cardiovascular risk and the mechanisms underlying this pathogenic link [75,76,77,78,79,80,81,82,83,84,85].

Adipokines	Pathogenic Mechanism
Leptin	Hyperleptinemia and leptin resistance leads to reduced energy consumption, hyperinsulinemia, and hyperlipidemia.
Adiponectin	Reduced plasma levels of adiponectin impair fatty acid oxidation and disrupt the body’s energy homeostasis.
Omentin	Enhances insulin action without affecting basal glucose transport.
Vaspin	Inhibits reactive oxygen species.
Apelin	Promotes cholesterol efflux.

## Data Availability

No new data were created or analyzed in this study. Data sharing is not applicable to this article.
